# Production of pazopanib hydrochloride nanoparticles (anti-kidney cancer drug) using a supercritical gas antisolvent (GAS) method

**DOI:** 10.1039/d4ra07079h

**Published:** 2024-12-18

**Authors:** Majid Bazaei, Bizhan Honarvar, Nadia Esfandiari, Seyed Ali Sajadian, Zahra Arab Aboosadi

**Affiliations:** a Department of Chemical Engineering, Marvdasht Branch, Islamic Azad University Marvdasht Iran honarvar2@gmail.com seyedali.sajadian@gmail.com; b Department of Chemical Engineering, Faculty of Engineering, University of Kashan Kashan 87317-53153 Iran

## Abstract

Supercritical fluid-based methods have been receiving increasing popularity in the production of pharmaceutical nanoparticles due to their ability to control the size and distribution of the particles and offer high purity products. The gas anti-solvent method is one of the methods in which a supercritical fluid serves as an anti-solvent. The aim of this work is to develop pazopanib hydrochloride nanoparticles as an anti-cancer agent by the supercritical GAS method. For this purpose, nanoparticles were produced at different temperatures (313, 323 and 333 K), pressures (10, 13 and 16 MPa), and initial solute concentrations (12, 22 and 32 mg ml^−1^) employing the Box–Behnken design. The results showed that pressure had the most significant effect on the particle size. The average initial particle size of unprocessed pazopanib hydrochloride was about 37.5 ± 8.7 μm. The optimum process parameter values were determined to obtain the smallest particle size using the BBD method. The parameters were optimized at 320 K, 16 MPa, and 12.6 mg ml^−1^. The average particle size was 311.1 nm, close to the predicted value of 302.3 nm. FTIR analysis indicated that the chemical structure remained unaltered. Furthermore, DSC and XRD results confirmed the reduction in particle size.

## Abstract 2

Supercritical fluid-based methods have been receiving increasing popularity in the production of pharmaceutical nanoparticles due to their ability to control the size and distribution of the particles and offer high purity products.

## Graphical abstract

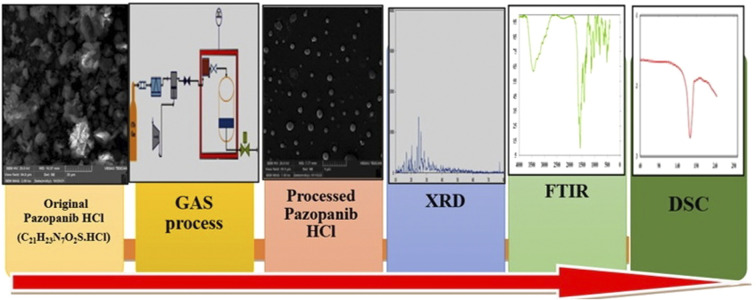

## 1 Introduction

The current advancements in the field of cancer treatment have made many cancers curable. The survival rate of kidney cancer patients is very high, and if this disease is diagnosed in time, it can be cured. However, if this cancer progresses and spreads to other organs, the treatment becomes more complicated and the probability of success decreases.^1^ Pazopanib hydrochloride (PAZ), with the molecular formula of C_21_H_23_N_7_O_2_S·HCl and molecular weight of 473.991 g mol^−1^, is one of the drugs for the treatment of kidney- and soft tissue-cancers. Known by the brand name of Votrient, PAZ slows down tumor growth by reducing its blood supply.^2^ This drug is used in the treatment of kidney cancer, soft tissue carcinoma and also in tumors that develop around muscles, joints, various body organs and blood vessels.^3^ PAZ has a low water solubility (about 0.0433 mg ml^−1^), categorizing it in class II of the biopharmaceutical classification system (BCS). At pH 1 (practically at pH > 4), it rarely dissolves in water. Its nanocrystallization, however, increases its dissolution and efficiency in the body.^4^ New methods have been developed for producing pharmaceutical particles on micro- or nano-scale with controlled particle size distribution and quality of crystals in terms of purity and geometric shape. Exploiting the special characteristics of supercritical fluids (SCFs), these methods are generally flexible, simple, and eco-friendly compared to other processes. The application of SCF as an alternative to the conventional precipitation process has been studied in the last two decades.^5–9^ Supercritical processes have many variations according to their operational goals. As mentioned above, the solubility of a medicinal substance in supercritical carbon dioxide (SC-CO_2_) is one of the effective parameters in choosing the production method of nanoparticles. One of the most important of these methods is the supercritical gas antisolvent (GAS), which is widely used in the pharmaceutical industry to produce small pharmaceutical particles. The use of SC-CO_2_ for the production of nanoparticles has been widely reported in the GAS process.^10–19^ Compared to other liquid solvents, SCFs allow the production of high purity materials, crystallization of materials, and production of small crystals. In the pharmaceutical industry, nano drugs offer high absorption, low side effects, and more effective performance. The production of uniform crystals in terms of particle size distribution is very important in injectable drugs. This technology is of great interest for hydrophobic drugs.^20,21^ One of the common fluids used in SCF technology is CO_2_, with a critical pressure of 7.38 MPa and a critical temperature of 304.2 K making it suitable for various processes. Therefore, it is possible to work with SC-CO_2_ near the ambient temperature, preventing the decomposition of temperature sensitive materials. Noteworthy, using SC-CO_2_ will eliminate or reduce the use of toxic or contaminated organic solvents. SC-CO_2_ can be easily separated from the resulting product by reducing the pressure. The high solubility of most organic solvents in supercritical fluids will result in the formation of a solvent-free product. In addition, carbon dioxide is a non-toxic, non-flammable, and low-cost liquid.^22–27^ The GAS process was first used for recrystallization. This method is especially useful for the crystallization of sensitive substances such as drugs, biological substances, and flammable substances at normal temperature. In the GAS method, high-pressure gas or SCF acts as an antisolvent for crystallizing or precipitation of a solid substance dissolved in an organic solvent. In this process, the antisolvent gas is highly soluble in the liquid solvent, causing volumetric expansion. Therefore, its density and solubility power decrease, resulting in the crystallization of the dissolved component. Most of the articles in this field address the effects of operating conditions, such as temperature, pressure, and concentration of the solute in the solution, on the size and size distribution of the formed particles, as listed in Table 1. The GAS process exploits the dissolution of gases in organic liquids to lower the solvation power of the liquid compared to the dissolved one, leading to the precipitation of the dissolved solid.^28,29^ The solid component is dissolved in the organic solvent until saturation, followed by exposure to the SCF under supercritical or near critical conditions. If the SCF dissolves well in an organic solvent (for example, CO_2_), the dissolved solid substance will be supersaturated when the SCF dissolves in the organic solvent, leading to the crystallization of this component. The main goal of this project was the laboratory production of PAZ nanoparticles by the GAS method for the first time. Given the solubility of this drug as measured in the previous work,^2^ it was decided to use the GAS method to produce PAZ pharmaceutical nanoparticles. For this purpose, operating conditions such as pressure (10, 13 and 16 MPa), temperature (313, 323 and 333 K), and concentration of solute (12, 22 and 32 mg ml^−1^) were assessed to produce more effective nanoparticles through the response surface method (RSM) with the help of Design Expert software. Finally, the shape, particle size, particle size distribution, purity, and nature of the particles were evaluated by X-ray diffraction (XRD), Fourier transform infrared spectroscopy (FTIR), differential scanning calorimetry (DSC), scanning electron microscopy (SEM), and dynamic light scattering (DLS) analyses.

**Table 1:** Some articles on the GAS method in the production of pharmaceutical particles

Drug	Method	Solvent	Particle size	Reference
Lysozyme	GAS	DMSO	180–300 nm	30
Paclitaxel	GAS	DMSO/H_2_O	117–200 nm	31
*Trans*-resveratrol	GAS	Acetone	21–32 nm	32
5-Fluorouracil	GAS	DMSO	260–600 nm	33
Crystalline β-carotene	GAS	DCM	0.5–5 μm	34
Cholesterol	GAS	Acetone	—	35
Paracetamol	GAS	Acetone	50 and 250 μm	36
Phenanthrene	GAS	Toluene	21–210 μm	37
Ginkgo-ginkgolides	GAS	Ethanol	0.8–240 μm	38
Beclomethason-17,21-dipropionate	GAS	Acetone	1.8–43.9 μm	39
Caffeine	GAS	Chloroform	26.3–128.3 μm	40
Carbamazepine	GAS	Acetone, ethyl acetate, DCM	50–285 nm	41
Theophylline	GAS	Ethanol	10–15 μm	42
Sulfamethoxazole	GAS	Acetone, methanol, ethanol	27–266 μm	43
Sertraline hydrochloride	GAS	DMSO	102–500 μm	44
Puerarin	GAS	Acetone, methanol, ethanol	29.7–49.26 μm	45
Posaconazole + 4-aminobenzoic acid	GAS	Acetonitrile	20–43 μm	46
Poly(ε-caprolactone)	GAS	Acetone	53–135 μm	47
Paracetamol	GAS	Ethanol, TEO	—	48
Naproxen + nicotinamide	GAS	Acetone	40–80 μm	49
Mefenamic acid + polyvinylpyrrolidone	GAS	Acetone, ethanol	—	50
Mefenamic acid + paracetamol	GAS	Acetone	1–350 μm	51
Liposome	GAS	Ethanol, chloroform	0.1–10 μm	52
Levothyroxine sodium	GAS	Ethanol	370–500 μm	53
Ketoconazole-4-aminobenzoic acid	GAS	Methanol, ethanol, acetone	12.8–14 μm	54
Itraconazole/l-malic acid	GAS	THF	—	55
Ibuprofen	GAS	Methanol, ethanol	1–3 μm	56
Griseofulvin	GAS	Dimethylformamide	0.5–500 μm	57
Gastroresitant	GAS	Acetone, DMSO	1–2 μm	58
5-Fluorouracil + nanoclay	GAS	Methanol	—	59
Finasteride	GAS	DMSO	333.56–1432.9 nm	60
Curcumin	GAS	Acetone, ethanol, acetonitrile, methanol	—	61
Copper indomethacin	GAS	DMSO, DMF, NMP	<100 μm	62
Cimetidine	GAS	Methanol, dichloromethane	3.1–26.7 μm	63
Carbamazepine–nicotinamide	GAS	Ethanol	—	64
Carbamazepine	GAS	Methanol	—	65
Capecitabine	GAS	DMSO	243.3–1090.9 nm	66
Aspirin	GAS	Methanol, acetone	48–124 μm	67
Tobramycin	GAS	Methanol	<500 nm	68
Hydrocortisone/PVP composites	GAS	Ethanol	—	69
Phentamidine and ethyl cellulose	GAS	Acetone, DMSO	0.5–1 μm	70
Lysozyme	GAS	DMSO	0.05–0.2 μm	71
l-Asparagi	GAS	C_2_H_6_O, H_2_O	20–200 μm	72
l-Ascorbic acid	GAS	C_2_H_6_O	<100 μm	72
C_60_(CO_2_)_0.95_	GAS	Toluene	1–70 μm	73
Sulphathiazole	GAS	Ethanol	6 μm	74
Polyamide	GAS	DMSO	1–10 μm	75
Barium chloride & ammonium chloride	GAS	DMSO	7–9 μm	76
			2–400 μm	
Cobalt chloride	GAS	Acetone	<300 μm	77
Phenanthrene	GAS	Toluene	150–550 μm	78
Glibenclamide	GAS	DMSO	99–386 nm	17

## 2 Experimental section

### 2.1. Materials

Pazopanib hydrochloride (CAS no.: 635702-64-6) was purchased as a solute with a minimum mass purity of 99.8% from Parsian Pharmaceutical Company (Karaj, Iran). Dimethyl sulfoxide (DMSO) (CAS no.: 67-68-5) solvent with a purity higher than 99.99% was purchased from Merck (Darmstadt, Germany), and the carbon dioxide (CAS no.: 124-38-9) as a supercritical fluid of industrial grade and 99.99% purity was purchased from Zagros (Shiraz, Iran). Table 2 shows the characteristics and molecular structures of the chemicals used.

**Table 2:** Characteristics and molecular structures of chemicals used

Compound	Formula	CAS number	Molecular structure	Mass fraction purity (%)	Analysis method
Pazopanib HCl	C_21_H_23_N_7_O_2_S·HCl	635702-64-6	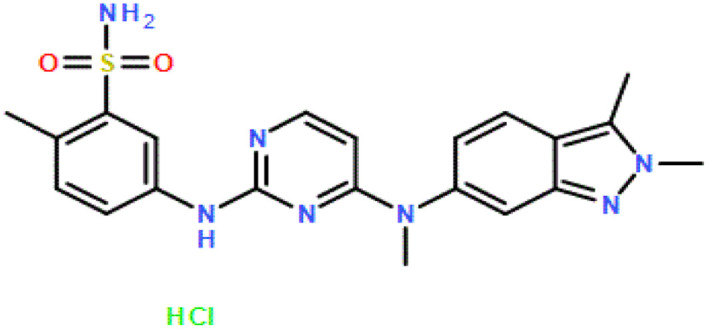	99.80%	HPLC^a^
DMSO	C_2_H_6_OS	67-68-5	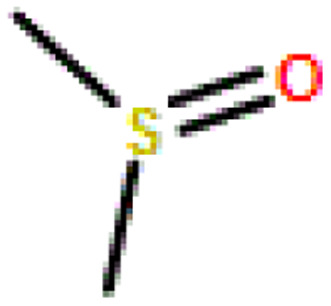	99.99%	GC^b^

a High-performance liquid chromatography.

b Gas chromatography.

### 2.2. Methods

#### 2.2.1. Experimental design (BBD)

The design of experiments (DOE) technique is a quality improvement technique. Using statistical experimental design methods can facilitate the design and production stages of new products and improve the existing ones. These principles have been used in most industries, such as the medical equipment, food, pharmaceutical, and chemical processing industries.^79^ Response surface design methods were first developed for chemistry, physics and biology. Owing to their reliable results, they are also used in the pharmaceutical industry. RSM, in short, involves the use of mathematical methods and statistical techniques to build experimental models. In these methods, in addition to the main effects between factors, it is possible to estimate interactive effects (quadratic) and interaction between factors (mutual effects). The response surfaces, or the local form of the response surfaces, are easily accessible and checked by examining the interactions between the factors. Box–Behnken design (BBD) and central composite designs (CCD) are some response surface design methods.^51,80,81^ In this article, the BBD experimental design method was used to produce PAZ pharmaceutical nanoparticles by the GAS method. As seen in Table 3, the effects of three effective parameters on the size of produced particles were studied (pressure (*X*_1_), temperature (*X*_2_) and initial concentration of solutes (*X*_3_)).

**Table 3:** Parameters and their various ranges for the purpose of BBD design

Coded levels	Pressure (*X*_1_, MPa)	Temperature (*X*_2_, K)	Solute concentration (*X*_3_, mg ml^−1^)
−1	10	313	12
0	13	323	22
+1	16	333	32

As shown in Table 3, the considered variables were designated as pressure (*X*_1_), temperature (*X*_2_), and solute concentration (*X*_3_) and examined at three levels coded as −1, 0, and 1, indicating high, intermediate and low values, respectively. For the sake of statistical calculations, the relationship between the coded and actual values was described as follows.1
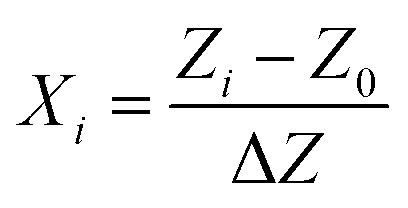
Here, *X*_*i*_ is the coded value of the respective variable, *Z*_*i*_ is the actual value corresponding to the *X*_*i*_, and *Z*_0_ denotes the actual value at the center point. Accordingly, Δ*Z* expresses the step change of the considered variable. The three variables were mathematically modeled using a quadratic polynomial model whose coefficients were calculated through a multiple regression analysis. The general form of this model is as follows.2
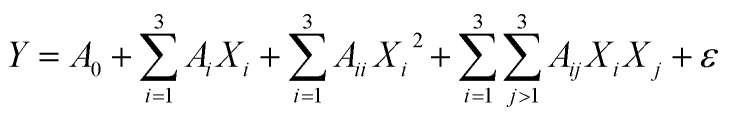
Here, *Y* represents the predicted response, *A*_0_ is a constant coefficient, *A*_*i*_ is the first-order linear coefficient, *A*_*ii*_ is the quadratic coefficient, *A*_*ij*_ is the coefficient of interaction, *X*_*i*_ and *X*_*j*_ are coded levels of the respective independent variable, and *ε* is the associated random error.^82^

#### 2.2.2. GAS process equipment

**Fig. 1:**
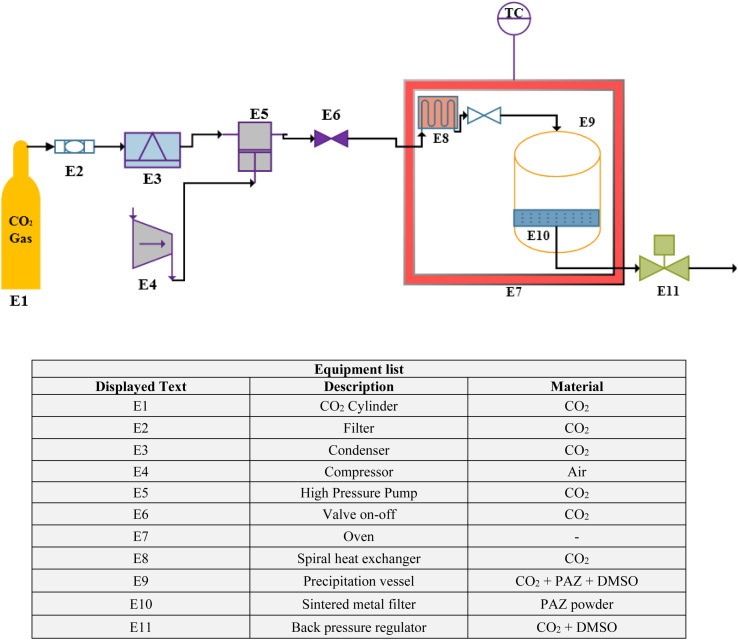
Schematic of the GAS process equipment.

A schematic of the GAS process equipment is shown in Fig. 1. CO_2_ was inside the cylinder (E1) and passed through a filter (pore size 1 μm) (E2). Then, the CO_2_ was liquefied by passing through the condenser (E3). Liquefied CO_2_ was pumped by a high pressure pump (type-CA 91502, Burbank, CA, USA) (E5). A valve on–off (E6) to control CO_2_ was located on the output flow from the pump. CO_2_ was heated by a spiral heat exchanger (E8). The rotary heat exchanger was located inside the oven (E7) and before the precipitation vessel (E9). The volume of the crystallizer was 100 ml. The pressure was adjusted by the back pressure regulating valve (type-1/4FNPT, Xi'an Shelok Instrument Technology Co.) (E11). The particles were collected on a sintered metal filter (E10) located at the end of the crystallizer.

#### 2.2.3. Experimental procedure

The desired substance for the production of nanoparticles (PAZ) was dissolved in DMSO. The initial concentration in the solvent was considered to be 12, 22 or 32 mg ml^−1^. 5 ml of this solution was sent into the crystallizer through a syringe. The oven was turned on and the temperature was 313, 323 or 333 K with an accuracy of ±0.1 K. CO_2_ was injected at a constant rate of 5 bar min^−1^ until the system reached the desired pressure (10, 13 or 16 MPa) with an accuracy of ±0.1 MPa. Then, the pump was turned off and the system was allowed to equilibrate for 60 min. After that, CO_2_ was introduced into the system so that the excess solvent was removed from the environment. In this case, it must be ensured that the system pressure is at the same pressure as before the pump was turned off, because, otherwise, there is a possibility that the precipitated materials will dissolve in the solvent from the decline of the pressure. The intensity of the constant flow of CO_2_ on the particles continued (4 g min^−1^ for 180 min) until all the solvent was removed from the system and the particles were dried. The particles settled on the filter at the end of the crystallizer. Finally, the pressure of the crystallizer decreased and reached ambient pressure. The crystallizer cell was opened and the particles were collected for FTIR, DLS, XRD, SEM and DSC analyses.

#### 2.2.4. Particle characterization

In this project, in accordance with studies conducted in the field of production of pharmaceutical nanoparticles by the GAS process, various devices were used to characterize the physical and chemical characteristics. Fourier transform infrared (FTIR) (Tensor II, Bruker Co., Germany) spectroscopy is based on the absorption of radiation and the investigation of vibrational mutations of molecules. This method was used to determine the structure and measure the chemical species based on spectra in the range 400–4000 cm^−1^.

In differential scanning calorimetry (DSC) (TA Co., USA), thermal analysis addresses the changes in material characteristics by temperature variations. By interpreting DSC results, a wide range of physical properties of materials can be determined. This analysis was conducted from ambient temperature up to 300 °C and at a maximum rate of 10 °C min^−1^.

Scanning electron microscopy (SEM) (MIRA III, TESCAN Co., Czech Republic) analysis was used for microstructural examinations to enlarge and analyze different parts of the samples. In this method, imaging is done with the help of electron beams (about 1–30 electron volts).

Dynamic light scattering (DLS) (SZ-100, Horiba Co., Japan) determines the size of particles and their distribution in liquids. However, for a wide range of applications, such as the pharmaceutical field, where it is necessary to disperse particles in a liquid, the behavior of particles in liquids should also be investigated. Therefore, 1 mg of produced PAZ nanoparticles was dissolved in 3 ml of deionized water and placed in a warm water bath (30 °C) for 10 minutes.

X-ray diffraction (XRD) (D8 ADVANCE, Bruker Co., Germany) is a rapid analytical method to identify the phase of a crystalline material. It can also offer some information on the chemical composition. This analysis was done in the angle range of 10° to 80°.

## 3 Results and discussion

### 3.1. Experimental design and checking the results of the Box–Behnken method

The goal of this project was to produce PAZ pharmaceutical nanoparticles. First, nanoparticles of this drug were prepared by the GAS method at different process conditions using the BBD response surface method. Based on this design, the average particle size (*X*_50_) was considered as the output variable (response) and the required number of experiments was found to be 15. Then experiments (L-15) were conducted using Design Export software 7.0.0 considering 3 parameters of temperature (313, 323 and 333 K), pressure (10, 13 and 16 MPa), and concentration of the solute (12, 22 and 32 mg ml^−1^) according to Table 4. The response relationships with different variables were determined by the BBD approach considering the coded factors using the multiple regression analysis of the experimental results. The relationship between the response and significant variables (pressure (*X*_1_), temperature (*X*_2_) and solute concentration (*X*_3_)) can be described using the following second-order quadratic polynomial equation.

3 *X*_50_ = 22.88 − 2.94*X*_1_ + 1.01*X*_2_ + 2.08*X*_3_ + 0.31*X*_1_*X*_2_ + 0.19*X*_1_*X*_3_ + 0.049*X*_2_*X*_3_ − 0.94*X*_1_^2^ + 1.08*X*_2_^2^ + 1.13*X*_3_^2^

**Table 4:** Operational conditions for the production of PAZ nanoparticles in this research with GAS process using the BBD method

Run	*X*_1_	*P* (MPa)	*X*_2_	*T* (K)	*X*_3_	Solute concentration (mg ml^−1^)	Mean particle size (*x*_50_ – nm)	Predicted value (nm)	Polydispersity index (PDI)
1	+1	16	+1	333	0	22	455.2	461.3	0.24
2	−1	10	+1	333	0	22	739.8	718.2	0.48
3	−1	10	−1	313	0	22	641.9	635.8	0.60
4	−1	10	0	323	+1	32	759.1	779.9	0.56
5	0	13	−1	313	+1	32	696.1	681.4	0.57
6	0	13	0	323	0	22	530.4	523.6	0.35
7	0	13	−1	313	−1	12	494.1	493.6	0.46
8	0	13	0	323	0	22	535.7	523.6	0.19
9	−1	10	0	323	−1	12	570.3	576.8	0.58
10	0	13	+1	333	+1	32	787.5	787.9	0.53
11	+1	16	0	323	+1	32	513.3	506.8	0.49
12	+1	16	0	323	−1	12	331.9	311.1	0.57
13	+1	16	−1	313	0	22	332.9	354.2	0.54
14	0	13	0	323	0	22	504.7	523.6	0.36
15	0	13	+1	333	−1	12	562.2	576.9	0.29

Analysis of variance (ANOVA) is widely used in hypothesis testing and statistical research. In this method, the difference between several statistical populations is analyzed. Due to the dispersion of the total data, it is possible to examine the variance between different groups. In this way, it is possible to test the average equality between various groups. Also, in regression models, the appropriateness of the model can be evaluated by decomposing the total variance into model variance and error variance. Therefore, the quadratic model is proposed by examining the regression models (linear, two-factor interaction (2FI), quadratic, and cubic) with the help of ANOVA analysis in Design Expert software 7.0.0 (Table 5). *R*^2^ (*R*-square), adjusted *R*^2^, and predicted *R*^2^ were used to check the fit of the quadratic model. The predicted residual error sum of squares (PRESS = 1607.45) presents a measure of model fit for the search points in the design and can be estimated by squaring the difference between the actual predicted values at each point and the sum of squares across the total set of points. Lower PRESS values represent a better model fit into the data points. As seen in Table 5, the values of *R*^2^ (*R*-square) (0.9875), adjusted *R*^2^ (0.9650), and predicted *R*^2^ (0.8577) indicated the suitability of the BBD model. Moreover, the ANOVA results in Table 6 show the validity of the *F*-value (more) and *P*-value (less). The parameters with a *p*-value below 0.05 have a significant effect on the production process with 95% confidence, while those with a *p*-value greater than 0.05 have a low impact. The high *F*-value indicates that the regression equations well express the response variations.^26,83,84^ Therefore, the parameters of temperature, pressure and initial concentration of the solution have the greatest effects on the particle size, according to the *F*-values in Table 6.

**Table 5:** ANOVA results

Source	Std dev.	*R*-Square	Adjusted *R*-square	Predicted *R*-square	Sequential *P*-value	Lack of fit *P*-value	PRESS
Linear	53.73	0.8843	0.8528	0.7717	<0.0001	0.0756	62675.46
2FI	62.71	0.8854	0.7995	0.4714	0.9942	0.0515	1.451 × 10^5^
**Quadratic**	**24.14**	**0.9894**	**0.9703**	**0.8577**	**0.0051**	**0.2692**	**39062.30**
Cubic	16.58	0.9980	0.9860		0.2692		

**Table 6:** Analysis of ANOVA for the production of PAZ pharmaceutical particles

Source	Sum of squares	df	Mean square	*F* value	*P*-value	Significance
Model	2.716 × 10^5^	9	30177.77	51.78	0.0002	Significant
*X*_1_ = pressure	1.452 × 10^5^	1	1.452 × 10^5^	249.16	<0.0001	Significant
*X*_2_ = temperature	18031.01	1	18031.01	30.94	0.0026	Significant
*X*_3_ = solute concentration	79520.72	1	79520.72	136.45	<0.0001	Significant
*X*_1_ *X*_2_	148.84	1	148.84	0.26	0.6348	No significance
*X*_1_ *X*_3_	13.69	1	13.69	0.023	0.8842	No significance
*X*_2_ *X*_3_	134.56	1	134.56	0.23	0.6512	No significance
*X*_1_^2^	4845.233	1	4845.233	8.31	0.0345	Significant
*X*_2_^2^	11199.71	1	11199.71	19.22	0.0071	Significant
*X*_3_^2^	11693.08	1	11693.08	20.06	0.0065	Significant
Residual	2913.93	5	582.786			
Lack of fit	2364.07	3	788.0233	2.87	0.2692	No significance
Pure error	549.86	2	274.93			
Cor. total	2.745 × 10^5^	14				

**Fig. 2:**
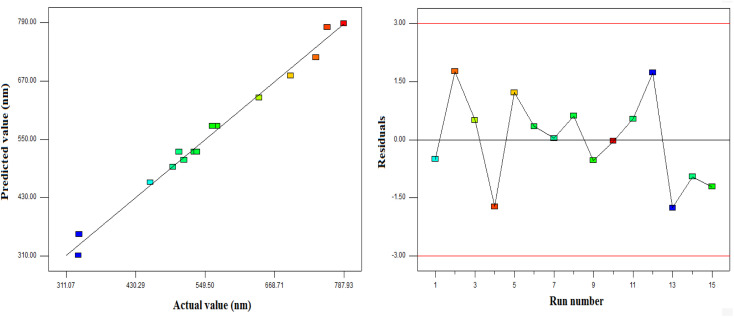
Diagnostic plots for model adequacy (model fitting) from the BBD method.

Fig. 2 presents the diagnostic plots of the model adequacy (model fitting) *via* the BBD method, which can be applied for comparison of the experimental results with the model calculated ones. As indicated in Fig. 2, the experimental results exhibited a significant proximity to the estimated data, implying good fitting to the empirical results. BBD was employed in the Design Expert software to optimize the operational conditions in order to minimize the PAZ particle size. According to this optimization, a temperature of 320 K, pressure of 16 MPa, and a solute (PAZ) concentration of 12.6 mg ml^−1^ were the optimum conditions.

**Fig. 3:**
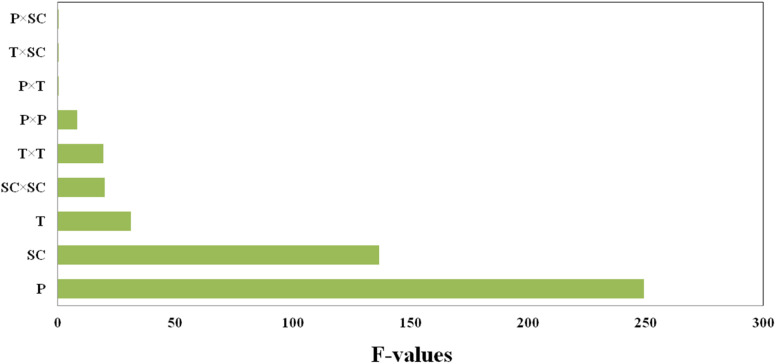
Pareto chart for parameters affecting the particle size of PAZ production (*P* = pressure (*X*_1_), *T* = temperature (*X*_2_) and SC = solute concentration (*X*_3_)).

A Pareto chart is used to highlight the most important factors. In the Pareto diagram (Fig. 3), it is clear that pressure has a significant impact (*p*-value < 0.0001) on the size of PAZ particles; initial concentration of the solution (*p*-value < 0.0001) and temperature (*p*-value = 0.0026) are placed in the next ranks. Moreover, according to Table 6, other parameters such as the quadratic effect of pressure (*X*_1_^2^) (*p*-value = 0.0345), the quadratic effect of temperature (*X*_2_^2^) (*p*-value = 0.0071), and the quadratic effect of the initial concentration of the solution (*X*_3_^2^) (*p*-value = 0.0065) show that all three parameters have a quadratic effect on the particle size. The effect of other interaction parameters on particle size, such as *X*_1_*X*_2_, *X*_1_*X*_3_, and *X*_2_*X*_3_, are almost the same and are not significant.

### 3.2. Investigating the effect of parameters on produced particles

Several parameters affect the quality and properties of the products, and also increase the yield and the performance of supercritical processes (GAS process), among which temperature, pressure, and initial concentration of the solution can be mentioned.

#### 3.2.1. The effect of pressure on the production of PAZ nanoparticles

**Fig. 4:**
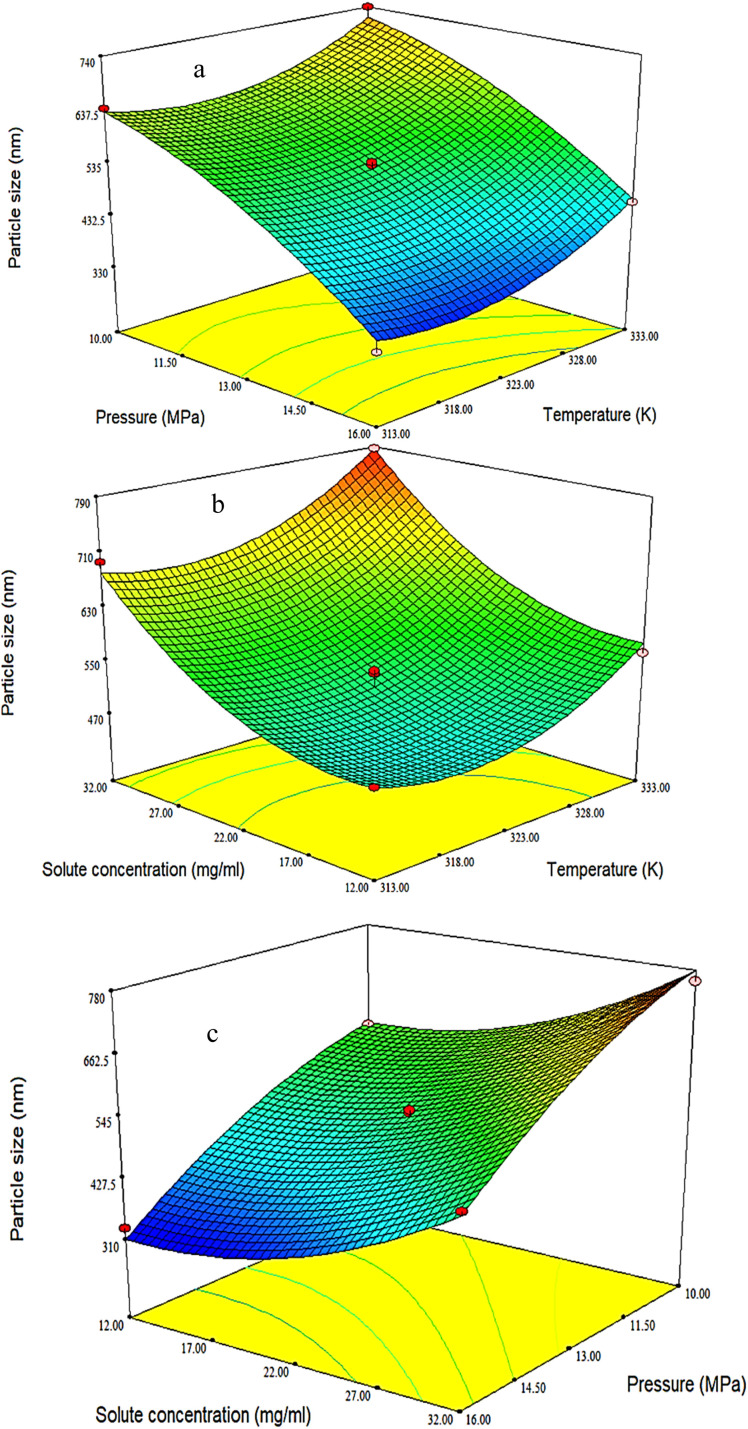
3D surface plots indicating the influences of (a) temperature and pressure on particle size (fixed parameter is solute concentration = 22 mg ml^−1^), (b) solute concentration and temperature on particle size (fixed parameter is pressure = 13 MPa), and (c) solute concentration and pressure (fixed parameter is temperature = 323 K) on particle size.

Pressure is one of the most important parameters in supercritical processes, including the GAS process, because it is one of the parameters that can be adjusted to optimize operational conditions. In general, the solubility of the anti-solvent in the organic solvent increases while the solubility of the dissolved solid decreases when raising the pressure at a constant temperature due to the volumetric expansion of the liquid phase. As a result, the solid starts to crystallize and smaller particles are produced.^5,67,85,86^ To check the pressure, experiments were carried out at three pressure levels (10, 13 and 16 MPa). According to Fig. 4a and Table 4, the average particle diameter was smaller when the pressure increased due to the dominance of the nucleation mechanism. It is also clear that the average particle size decreases with increasing pressure. Similar results were obtained by Chen *et al.*,^38^ for the preparation of ginkgo medicinal substance, Domingo *et al.*^87^ for the production of ultrafine organic crystal particles, Park *et al.*^88^ for the production of nanoparticles of sulfa drugs, and Esfandiari *et al.*,^5^ for the production of fine pharmaceutical particles of ampicillin. All the mentioned researchers showed that smaller particles were obtained at higher pressures.

#### 3.2.2. The effect of temperature on the production of PAZ nanoparticles

Temperature affects the volumetric expansion of the solvent, which ultimately influences the shape and physical properties of the products. At lower temperatures (and constant other operational conditions), it is finer. The solubility of the solid substance in the solvent also increases with temperature enhancement at a constant pressure, which leads to crystallization at a lower temperature to increase the production rate of a higher quality product.^33,39,89^ In this project, the effect of temperature on the production of PAZ nanoparticles by the GAS method was addressed. While changing the temperature to three levels (313, 323 and 333 K), the particle size and particle size distribution were studied. The size distribution of particles and average particle size are shown in Fig. 4b and Table 4. As can be seen, temperature elevation in the precipitation process increased the average particle size of PAZ. The solubility of many medicinal substances in organic solvents is proportional to temperature. In many pharmaceuticals, solubility increases with increasing temperature and reduction of particle size by gas process is based on decreasing solubility. Therefore, the rate of supersaturation and nucleation can be controlled by temperature. Ion *et al.*^90^ and Wichianphong *et al.*^51^ reported similar results regarding the deposition of other substances (mefenamic acid–paracetamol).

#### 3.2.3. The effect of initial solute concentration on the production of PAZ nanoparticles

The crystal size strongly depends on the initial concentration of the solid dissolved in the solvent (at the same pressure and temperature). At a higher initial concentration of the solid substance, the size of the crystals will be larger and their density will be higher.^85,91^ To investigate the effect of initial concentration of the solution on the particle size distribution of PAZ, the initial concentration of the solution was changed at three levels (12, 22 and 32 mg ml^−1^). Fig. 4c shows the variation of average particle size with solute concentration. As can be seen in Fig. 4c and Table 4, larger particles were formed by increasing the concentration of the solute. Nucleation occurs at a smaller volume ratio by raising the initial solute concentration. Therefore, the particles have more time to grow, leading to larger particles.^92–95^ Ardestani *et al.*^6^ for the preparation of phthalocyanine green particles, and Najafi *et al.*^10^ for the production of rosuvastatin calcium nanoparticles, reported similar results.

### 3.3. Investigating the characteristics of produced PAZ nanoparticles

#### 3.3.1. SEM and DLS analysis results of PAZ particles

**Fig. 5:**
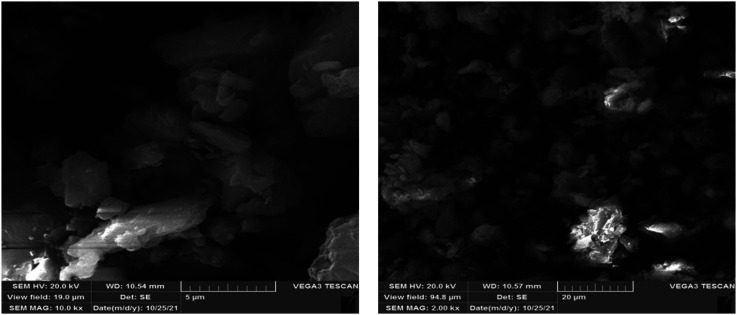
SEM image of the original PAZ drug.

As seen in Fig. 5, the average size of the original PAZ drug particles was about 37.5 ± 8.7 μm and they had an irregular and polyhedral shape. To calculate the average particle size (37.5 ± 8.7 μm), the sizes of about 50 particles were randomly measured. Fig. 5 shows the SEM images of the original PAZ drug particles before the GAS process.

**Fig. 6:**
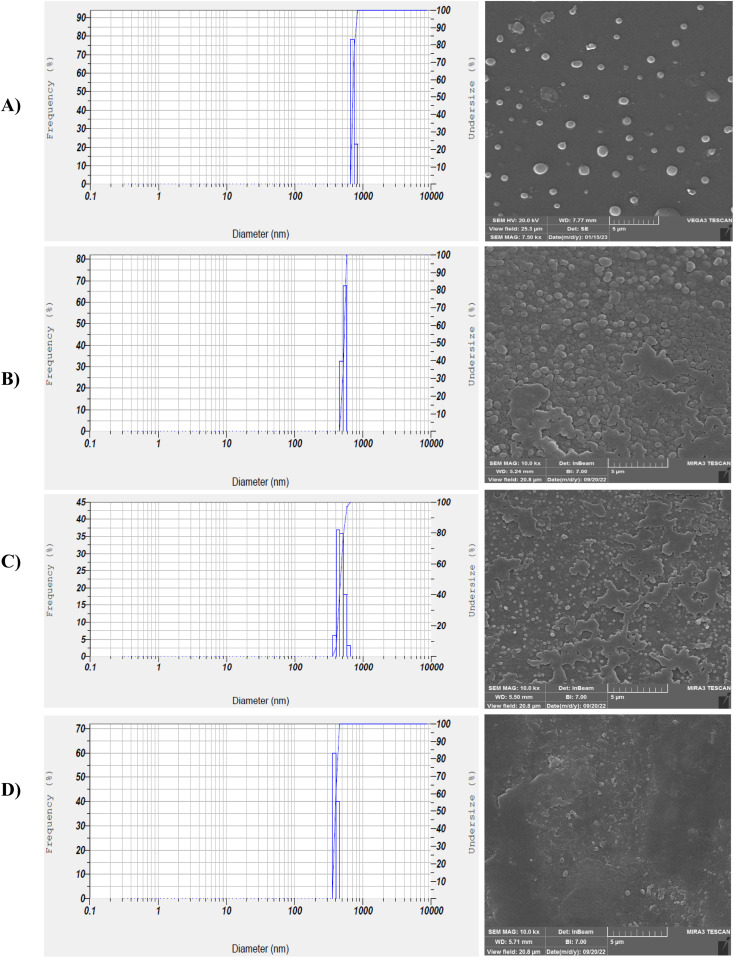
SEM images and DLS results (according to Table 4): (A) run 2, (B) run 15, (C) run 1, (D) run 13 (optimum conditions).

Fig. 6 depicts the structure and morphology of PAZ nanoparticles obtained (SEM analysis) under different conditions. Table 4 also lists the size distribution of precipitated particles (DLS analysis). The morphology of GAS-processed particles altered from irregular to spherical at higher pressures. With increasing pressure, the nucleation mechanism prevails, so smaller particles are observed. Fig. 6 shows the DLS diagrams (run 1, 2, 13, and 15), which are clearly consistent with the SEM results. Accordingly, the smallest particles were reached at the highest pressure (16 MPa). The particle sizes of PAZ nanoparticles ranged from 330 to 788 nm.

#### 3.3.2. FTIR analysis result of PAZ particles

**Fig. 7:**
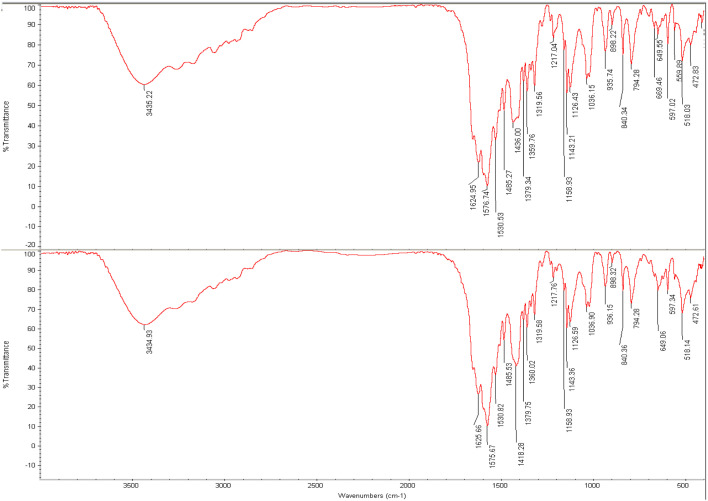
FTIR analysis of (a) original PAZ (top) and (b) processed PAZ (at optimum conditions) (bottom).

This method can also identify organic compounds, because the spectra of these compounds are usually complex and have a number of maximum and minimum peaks that can be used for comparative purposes.^96^ The measured FTIR spectra, on KBr pellets, within the frequency range of 400–4000 cm^−1^ are shown in Fig. 7. Fig. 7A and B present the FTIR spectra of the un-processed and processed PAZ samples, respectively. The FTIR spectra exhibit various absorption bands, of which the primary ones are considered herein. As shown in Fig. 7, there is no significant difference in shape and position between the absorption peaks obtained for the two samples (before and after process). As can be seen, the structure of this material did not change during the process. Similar results were found by Ramana *et al.*,^97^ Herbrink *et al.*,^98^ and Nadaf *et al.*.^99^ The main peaks were identified by FTIR analysis and are listed in Table 7.

**Table 7:** FTIR spectra for various bonds of PAZ

Chemical functional bond	Original PAZ	Processed PAZ
Stretching bond –NH	3435.22	3434.93
Bending bond –CH	1576.74	1575.67
Bending bond –CH_3_	1485.27	1485.53
Stretching bond –C–N	1143.21	1143.36
Stretching bond –S–N	1036.15	1036.90

#### 3.3.3. XRD analysis result of PAZ particles

**Fig. 8:**
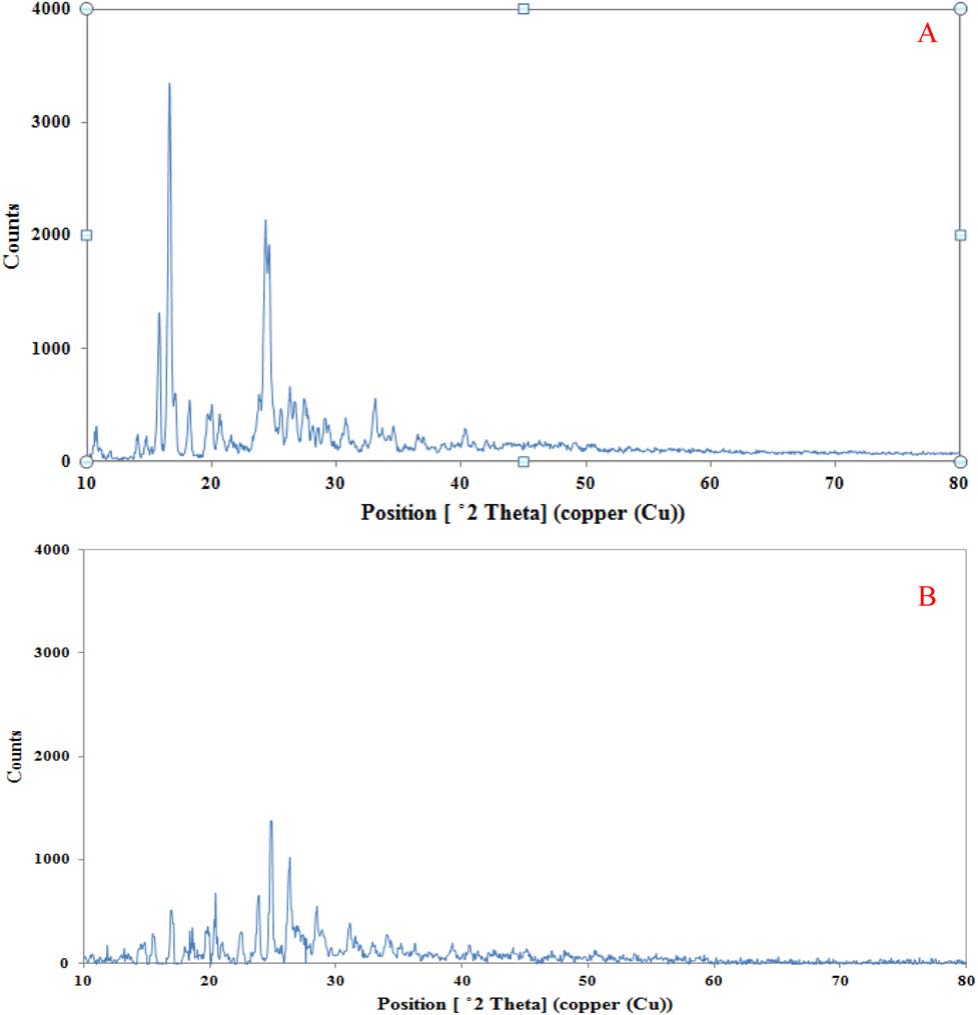
XRD spectra of (A) original PAZ and (B) processed PAZ (optimum conditions).

XRD analysis was performed on the original PAZ particles and the sample obtained from the GAS process to compare their crystalline properties. Fig. 8 shows the XRD patterns of the original and the GAS-processed particles. The XRD patterns of the original and GAS-processed particles show that the structure of the particles remained intact. Therefore, the PAZ particles obtained from the GAS process maintained their crystalline structure. However, the intensity of the peaks related to the produced PAZ particles is smaller than that of the original PAZ particles, which can be assigned to two factors: the decrease in the crystallinity degree of the produced sample and the decline of the particle size. Similar results can be found in the articles of Kaduk *et al.*,^100^ and Herbrink *et al.*^101^

#### 3.3.4. DSC analysis result of PAZ particles

**Fig. 9:**
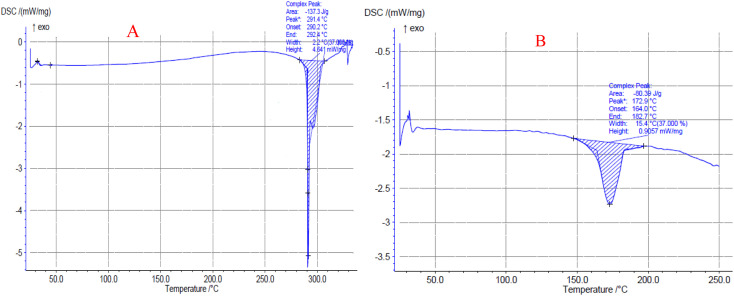
DSC curves of (A) original PAZ and (B) processed PAZ (optimum conditions).

In Fig. 9A and B, the thermal behavior of original PAZ and PAZ nanoparticles produced by GAS method is shown. According to Fig. 9A, the melting point peak is at 291.4 °C, which corresponds to the results of Shen *et al.*^102^Fig. 9B also indicates that PAZ nanoparticles (melting point peak at 172.9 °C) show a similar thermal behavior compared to the original PAZ particles. The decrease in the melting point could be due to the decrement in the degree of crystallinity of the nanoparticles and the decrease in the size of the particles, which confirms the results of XRD. Table 8 lists the melting points and enthalpies of original PAZ and PAZ nanoparticles. Based on the values of normal enthalpy change of original PAZ and PAZ particles, the crystalline degree of the nanoparticles is 59.3%. Similar results can be observed in Herbrink *et al.*^98^

**Table 8:** Values of melting temperature and enthalpy change of original PAZ and PAZ nanoparticles

Item	Enthalpy change Δ*H* (J g^−1^)	Onset (°C)	Peak (°C)	End set (°C)
Original PAZ	137.3	290.2	291.4	292.4
PAZ nanoparticle	80.39	164.0	172.9	182.7

#### 3.3.5. Optimum conditions

The optimum values of the process parameters were determined to obtain the smallest particle size using the BBD method implemented in the Design Expert software. These values were determined to be a temperature of 320 K, a pressure of 16 MPa, and a solute concentration of 12.6 mg ml^−1^. These values were predicted to yield particles of 302.3 nm in size. The BBD method was used to evaluate the accuracy and validity of the optimization method through experiments, and the average particle size was found to be 311.1 nm, which was very close to the predicted value.

## 4 Conclusion

The production of PAZ nanoparticles with a GAS process at different operational conditions has been rarely addressed. Thus, this article addresses the comprehensive identification of changes in the physical and chemical properties of PAZ under GAS process. Examining the results of PAZ micronization by GAS process in the current research shows that the size of the drug particles decreased from 37.5 ± 8.7 μm to 330–788 nm. Operating parameters (among the various parameters) for the production of PAZ nanoparticles are pressure (10, 13 and 16 MPa), temperature (313, 323 and 333 K) and solute concentration in the initial solution (12, 22 and 32 mg ml^−1^). Based on the Box–Behnken experimental design (BBD), the smallest pazopanib hydrochloride particles were produced at 320 K, 16 MPa and a solution concentration of 12.6 mg ml^−1^. Examination of the chemical structure of nanoparticles by FTIR showed that the structure of this material did not change during the process. The XRD patterns of the original and GAS-processed particles showed that the structure of the particles remained intact. Examination of the thermal behavior of the nanoparticles reveals a decline in the melting point of nanoparticles by 118.5 °C compared to that of the original drug. Furthermore, the degree of crystallinity of the nanoparticles decreased.

## Nomenclature

*P*
: Pressure
*T*
: Temperature
SC: Solute concentration
*H*
: Enthalpy
*X*_1_, *X*_2_, *X*_3_: Parameters of BBD design

### Greek letters

Δ: Property change

### Abbreviations

ANOVA: Analysis of variance
BBD: Box–Behnken design
BCS: Biopharmaceutical classification system
CCD: Central composite design
DMSO: Dimethyl sulfoxide
DCM: Dichloromethane
DLS: Dynamic light scattering
DSC: Differential scanning calorimetry
DOE: Design of experiments
df: Differential
FTIR: Fourier transform infrared
GAS: Gas anti solvent
PAZ: Pazopanib hydrochloride
RSM: Response surface method
XRD: X-ray diffraction
Std dev.: Standard division
SEM: Scanning electron microscope
SC-CO_2_: Supercritical carbon dioxide
SCF: Supercritical fluid

## Data availability

The authors confirm that the data supporting the findings of this study are available within the article.

## Author contributions

M. B.: writing original draft, investigation, software, formal analysis, data curation. B. H.: methodology, supervision, conceptualization, review and editing. N. E.: investigation, validation, methodology, conceptualization, and review and editing. S. A. S.: investigation, conceptualization, project administration, and review and editing. Z. A. A.: methodology, validation, and investigation.

## Conflicts of interest

The authors declare that they have no known competing financial interests or personal relationships that could have appeared to influence the work reported in this paper.
